# cAMP controls the restoration of endothelial barrier function after thrombin‐induced hyperpermeability via Rac1 activation

**DOI:** 10.14814/phy2.12175

**Published:** 2014-10-24

**Authors:** Muhammad Aslam, Christian Tanislav, Christian Troidl, Rainer Schulz, Christian Hamm, Dursun Gündüz

**Affiliations:** 1 Department of Cardiology and Angiology, Justus Liebig University, Giessen, Germany; 2 Department of Neurology, Justus Liebig University, Giessen, Germany; 3 Institute of Physiology, Justus Liebig University, Giessen, Germany

**Keywords:** Adenylyl cyclase, adherens junctions, calcium, Rac1, RhoA

## Abstract

Inflammatory mediators like thrombin disrupt endothelial adherens junctions (AJs) and barrier integrity leading to oedema formation followed by resealing of AJs and a slow recovery of the barrier function. The molecular mechanisms of this process have not yet been fully delineated. The aim of the present study was to analyse the molecular mechanism of endothelial barrier recovery and thrombin was used as model inflammatory mediator. Thrombin caused a strong increase in endothelial permeability within 10 min accompanied by loss of Rac1 but not cdc42 activity, drop in cellular cAMP contents, and a strong activation of the endothelial contractile machinery mainly via RhoA/Rock signalling. Activation of RhoA/Rock signalling precedes and is dependent upon a rise in the cytosolic Ca^2+^ concentration. Inhibition of cytosolic Ca^2+^ rise but not MLCK or Rock enhances the recovery of endothelial barrier function. The cellular cAMP contents increased gradually during the barrier recovery phase (30–60 min after thrombin challenge) accompanied by an increase in Rac1 activity. Inhibition of Rac1 activity using a specific pharmacological inhibitor (NSC23766) abrogated the endothelial barrier recovery process, suggesting a Rac1‐dependent phenomenon. Likewise, inhibition of either adenylyl cyclase or the cAMP‐effectors PKA and Epac (with PKI and ESI‐09, respectively) caused an abrogation of Rac1 activation, resealing of endothelial AJs and recovery of endothelial barrier function. The data demonstrate that endothelial barrier recovery after thrombin challenge is regulated by Rac1 GTPase activation. This Rac1 activation is due to increased levels of cellular cAMP and activation of downstream signalling during the barrier recovery phase.

## Abstract (precis)

e12175

In the present study, we analysed the changes in the dynamic activities of members of the Rho family of GTPases and the role of endogenous cAMP signalling in the restoration of thrombin‐induced EC hyperpermeability. To imitate the in vivo conditions, the thrombin was present during whole experiments. The study demonstrates that challenging the human umbilical vein endothelial cell (HUVEC) monolayers with thrombin results in a prompt activation (within first 10 min) of RhoA/Rock signalling and inhibition of Rac1 activity accompanied by a reduction in cellular cAMP contents. During the recovery phase of EC barrier function (30–60 min), an activation of Rac1 but not cdc42 occurs which is accompanied by an increase in intracellular levels of cAMP. Inhibition of adenylyl cyclase (AC) or downstream cAMP signalling abrogates Rac1 activation during the recovery phase and impedes the restoration of EC barrier function.

## Introduction

Vascular endothelium (VE) forms a semi‐selective barrier and thus regulates the transport of macromolecules and blood cells across the vessel wall (Mehta and Malik 2006). The integrity of the endothelial cell (EC) barrier function is regulated by the actin‐myosin based contractile machinery and actin cytoskeleton‐anchored adherens junctions (AJs) consisting of VE‐cadherin and catenins linked to the actin cytoskeleton (Dejana et al. 2008). Exposure of ECs to inflammatory mediators like thrombin results in the activation of multiple signalling pathways, finally leading to EC barrier failure (Schnittler et al. 1990; Rabiet et al. 1996). Thrombin is generated in the vessels from circulatory prothrombin and not only regulates blood coagulation but also plays a major role in the development of pulmonary and brain oedema (Bogatcheva et al. 2002; Mehta and Malik 2006). Thrombin induces the activation of EC contractile machinery via increased intracellular levels of Ca^2+^, activation of myosin light chain kinase (MLCK) and RhoA/Rock signalling which downstream inhibits the myosin light chain phosphatase (MLCP) (van Hinsbergh 1997). Thrombin also inhibits the activation of Rac1 (Aslam et al. 2012) leading to disruption of AJs and EC barrier integrity. The thrombin‐induced hyperpermeability is transient and ECs reseal within 1–2 h after thrombin challenge. Immediately after the barrier disrupting effect of thrombin, recovery of the EC barrier function starts. The mechanisms of EC barrier restoration are critical for the maintenance of basal permeability, yet our understandings of recovery of EC barrier function in response to the permeability increasing mediators have not been well delineated. The central role is played by the dynamic activities of the Rho family of GTPases (Mehta and Malik 2006).

In the present study, we analysed the changes in the dynamic activities of members of the Rho family of GTPases and the role of endogenous cAMP signalling in the restoration of thrombin‐induced EC hyperpermeability. To imitate the in vivo conditions, the thrombin was present during whole experiments. The study demonstrates that challenging the human umbilical vein endothelial cell (HUVEC) monolayers with thrombin results in a prompt activation (within first 10 min) of RhoA/Rock signalling and inhibition of Rac1 activity accompanied by a reduction in cellular cAMP contents. During the recovery phase of EC barrier function (30–60 min), an activation of Rac1 but not cdc42 occurs which is accompanied by an increase in intracellular levels of cAMP. Inhibition of adenylyl cyclase (AC) or downstream cAMP signalling abrogates Rac1 activation during the recovery phase and impedes the restoration of EC barrier function.

## Materials and Methods

### Materials

HRP‐conjugated anti‐mouse IgG, and rabbit IgG antibodies were purchased from Amersham Biosciences (Heidelberg, Germany); human thrombin from Behring (Marburg, Germany); anti VE‐cadherin (clone TEA 1, mouse IgG) from Beckman Coulter (Krefeld, Germany); ESI‐09 from Biolog (Bremen, Germany); benzonase, forskolin, myristoylated PKI, and Y‐27632, from Calbiochem (Darmstadt, Germany); anti‐phospho‐MLC and anti‐GAPDH antibodies from Cell signaling (Danvers, MA); Rac1 activation assay kit from cytoskeleton (Denver); Pierce^®^ ECL solution from Fischer Scientific (Niederlassung Nidderau, Germany); Alexa‐Flour labelled anti‐mouse IgG and anti‐rabbit IgG antibodies from Invitrogen (Karlsruhe, Germany); anti‐Rac1‐GTP antibody from NewEast Biosciences (King of Prussia, PA); EC basal medium plus supplement pack from PromoCell (Heidelberg, Germany); Complete^®^ protease inhibitor cocktail from Roche (Mannheim, Germany); 3‐isobutyl‐1‐methylxanthine (IBMX), Phalloidin‐TRITC, L‐NAME, L‐NNA, MDL‐12 330A, and anti‐vinculin (clone hVIN‐1, mouse IgG) from Sigma (Steinheim, Germany); anti‐phospho‐MYPT1 (Thr850) from Merck Millipore (Schwalbach, Germany), and Transwell polycarbonate membrane filters (24‐mm round) were from Greiner Bio‐One (Frickenhausen, Germany). All other chemicals were of the best available quality, usually analytical grade.

### Cell culture

The study conforms to the principles outlined in the “Declaration of Helsinki” (*Cardiovascular Research* 1997; 35: 2–3). HUVEC were isolated from umbilical cords derived from normal healthy uncomplicated pregnancies obtained from university hospital Giessen after approval from hospital ethics committee and cultured as described before (Aslam et al. 2010). The experiments were in HUVECs performed from passages 1–2.

### Experimental protocols

The basal medium used in incubations was modified Tyrode's solution (composition in mmol/L: 150 NaCl, 2.7 KCl, 1.2 KH_2_PO_4_, 1.2 MgSO_4_, 1.0 CaCl_2_, and 30.0 *N*‐2‐hydroxyethylpiperazine‐*N*′‐2‐ethanesulfonic acid; pH 7.4, 37°C). Agents were added as indicated. Stock solution of myristoylated PKI, thrombin, and Y27632 were prepared immediately before use with basal medium. Appropriate volumes of these solutions were added to the cells yielding final solvent concentrations < 0.1% (vol/vol). The same final concentrations of basal medium were included in all respective control experiments. Cells were incubated with serum free Tyrode's solution 30 min prior to adding of drugs. In experiments where pharmacological inhibitors were used, the inhibitors were added 30 min before adding thrombin. In sets of experiments, where PKI was used, cells were preincubated with PKI for 60 min followed by the addition of relevant stimulators as stated in the figure legends.

In a set of pilot experiments concentration‐response relationships were determined to find the optimal effective drug concentration: thrombin (0.3 IU/mL), ESI‐09 (3 *μ*mol/L), MDL‐12 330A (10 and 25 *μ*mol/L), myristoylated PKI (20 *μ*mol/L), Y‐27632 (10 *μ*mol/L).

### Recovery experiments

*Thrombin was used to induce the* EC monolayers hyperpermeability or activate EC barrier disrupting signalling. In order to mimic the in vivo conditions, thrombin was present during whole of the experiment. The measurements were made at various time points after adding the thrombin. The time taken to induce maximum hyperpermeability was considered as barrier disrupting phase while time taken by EC monolayers from maximum permeability back to basal level as recovery phase. In experiments where cytochalasin D was used, the cells were treated with cytochalasin for 10 min and then medium was changed to wash the cytochalasin off and the cells were allowed to recover for indicated time periods.

### Loading of BAPTA‐AM

EC monolayers cultured on either filter membranes or a cell culture dish were incubated with 10 *μ*mol/L 1,2‐bis(2‐aminophenoxy)ethane‐*N*,*N*,*N*8,*N*8‐tetraacetic acid‐AM (acetoxymethyl ester of BAPTA) in Opti‐Mem medium (Invitrogen) supplemented with 2% (vol/vol) heat‐inactivated foetal calf serum (FCS) for 30 min at 37°C. After the incubation period, the extracellular BAPTA‐AM was removed by medium change and the experiments were started.

### Western blotting

Western blotting was performed as described previously (Aslam et al. 2010). Proteins were detected by FUSION‐FX7 (PeqLab) system and images were analysed using Quantity One software (Bio‐Rad).

### Rac1/cdc42 pulldown assay

The activation of Rac1 was assessed by pull‐down assay. ECs were washed with ice‐cold PBS and lysed with 600 *μ*L of lysis buffer on ice for 10 min. Lysate was centrifuged at 14,000×g for 1 min at 4°C. 500 *μ*g of cell lysates were incubated with 10 *μ*g of GST‐PAK beads (Cytoskeleton Inc., Denver, CO) at 4°C for 40 min. The beads were washed four times with wash buffer, heated to 95° for 5 min with 30 *μ*L of Laemmli buffer and loaded on 12.5% SDS gel. Bound Rac1 or cdc42 protein was then detected by immunoblotting using polyclonal antibodies against Rac1 or cdc42 (Cell Signaling Inc., Danvers, MA), respectively. The total amount of Rac1 or cdc42 in cell lysates were used as a control for the cross‐comparison of Rac1 or cdc42 activities (level of GTP‐bound Rac1).

### Macromolecule permeability measurement

The permeability of trypan blue labelled albumin across HUVEC monolayers was studied in a two‐compartment system separated by a filter membrane as described previously (Pfeil et al. 2009). Briefly, both compartments contained as basal medium modified Tyrode's solution (composition in mmol/L: 150 NaCl, 2.7 KCl, 1.2 KH_2_PO_4_, 1.2 MgSO_4_, 1.0 CaCl_2_, and 30.0 N‐2‐hydroxyethylpiperazine‐N′‐2‐ethanesulfonic acid; pH 7.4, 37°C) supplemented with 2% (vol/vol) normal calf serum. There was no hydrostatic pressure gradient between both compartments. The “luminal” compartment containing the monolayer had a volume of 2.5 mL, and the “abluminal” of 6.5 mL. The fluid in the “abluminal” compartment was constantly stirred. Trypan blue‐labelled albumin (60 *μ*mol/L) was added to the luminal compartment. The appearance of the labelled albumin in the abluminal compartment was continuously monitored by pumping the liquid through a spectrophotometer (Specord 10, Zeiss, Jena, Germany). Any increase in the concentration of labelled albumin was detected with a time delay of less than 15 sec.

### Cytosolic Ca^2+^ measurement

Free cytosolic [Ca^2+^]_i_ levels were determined using the fluorescent Ca^2+^ indicator fura‐2. Non confluent EC monolayers cultured on 20‐mm glass coverslips were incubated with 5 *μ*mol/L fura 2‐AM in Opti‐Mem medium (Invitrogen) supplemented with 2% (vol/vol) heat inactivated FCS at 37°C in the dark. After a 45‐min incubation period, the extracellular fura 2‐AM was removed by medium change followed by a 20‐min incubation period in the same medium before measurements were started. The coverslips were then mounted in a fluorescence microscope (IX 70; Olympus, Hamburg, Germany). [Ca^2+^]_i_ was analysed using a TILL Photonics (Martinsried, Germany) imaging system. During incubations, the excitation wavelength was alternated between 340 and 380 nm (bandwidth of 8 nm).

### cAMP assay

Human umbilical vein endothelial cell were cultured in six‐well plates until confluence, washed once with modified Tyrodes’ buffer supplemented with 100 *μ*mol/L IBMX, a phosphodiesterase inhibitor, to prevent the hydrolysis of cAMP, then were incubated in the same solution for 30 min in the presence or absence of the other reagents. After the incubation cAMP content of each well was determined by a acetylated version of colorimetric immunoassay method according to the manufacturer's instructions (Assay Designs, Loerrach, Germany) using the “Infinite® 200” fluorescent plate reader (Tecan, Austria). Forskolin (10 *μ*mol/L) was included as positive control on each plate. Three measurements per treatment group were performed on cells from an individual culture and averaged to yield one value. Negative controls omitting primary antibody but adding secondary antibody were included in all experiments. Experiments were done on three individual cultures.

### Immunocytochemistry and confocal microscopy

Human umbilical vein endothelial cell were grown until confluence on glass cover slips. After treatment cells were washed with PBS, fixed with 4% PFA at 37°C for 20 min, and permeabilised with 0.2% Triton X‐100. Nonspecific binding was blocked by incubating cells with blocking solution (5% bovine serum albumin [BSA] + 5% neonatal calf serum [NCS] for 1 h. Cells were incubated with the primary antibody (e.g., anti active Rac1‐GTP; 1:100) overnight at 4°C, washed three times with PBS, and subsequently incubated with the secondary antibody (1:1000) for 1 h at room temperature. For actin cells were stained with phalloidine‐TRITC (1:50) for 1 h at room temperature. The cover slips were embedded in fluorescent mounting medium (CitiFluor, UK) and put onto glass objective slides. Images were obtained using a Zeiss LSM 510 META (Zeiss; Jena, Germany) confocal microscope.

### Statistical analysis

Data are given as means ± SEM of *n* experiments using independent cell preparations. The comparison of means between groups was performed by one‐way analysis of variance (ANOVA) followed by a Student‐Newman‐Keuls post‐hoc test using graphpad prism software (V 6.0; Graphpad Inc. La Jolla). Probability (*P*) values of less than 0.05 were considered significant.

## Results

### Dynamics of endothelial permeability, actin cytoskeleton, and AJs after thrombin challenge

**Figure 1.**
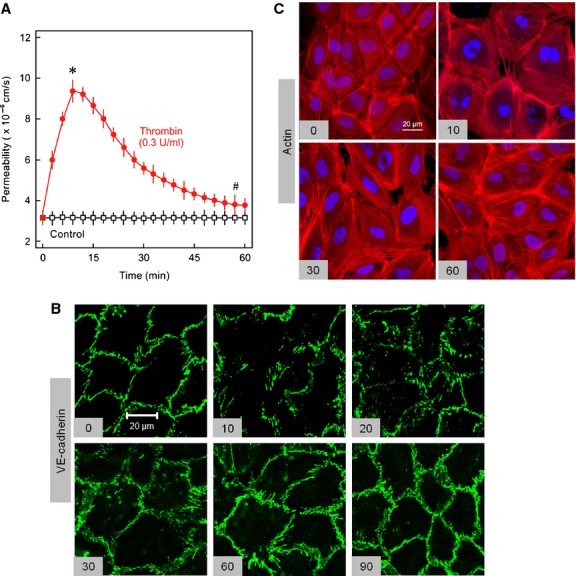
Dynamics of endothelial permeability, actin cytoskeleton, and AJs after thrombin challenge. (A) EC monolayers were exposed to thrombin (Thr; 0.3 IU/mL) or vehicle (control) as indicated and albumin flux (permeability) was measured as described in methods section. Mean ± SEM of three experiments of independent cell preparations, *P <**0.05 versus control. (B) EC monolayers were exposed to thrombin (Thr; 0.3 IU/mL) for different time points (min) as indicated or vehicle, methanol fixed, and immunostained for VE‐cadherin or (C) paraformaldehyde (4%) fixed and stained with phalloidin‐TRITC for actin visualisation. Representative figures of three experiments of independent cell preparation.

Endothelial barrier function was analysed by measuring the rate of flux of trypan blue‐labelled albumin across HUVEC monolayers. As shown in Fig. 1A thrombin caused a prompt increase in endothelial permeability reaching maximum within 10 min and slowly returned to basal level within approx. 60 min. The dynamics of increase in permeability are well co‐ordinated with the changes in VE‐cadherin localisation at cell‐cell junctions (Fig. 1B) and actin cytoskeleton (Fig. 1C). Under basal conditions VE‐cadherin and most of the filamentous actin (F‐actin) are localised at cell‐cell junctions. Thrombin, within 10 min, causes loss of peripheral actin localisation accompanied by enhanced actin stress fibres and disappearance of VE‐cadherin from the junctions and an increase in permeability. This is followed by slow disappearance of actin stress fibres and reappearance of VE‐cadherin and peripheral actin at the cell‐cell junctions which is almost accomplished during 60 min.

### Dynamics of activities of Rho GTPases, endothelial contractile machinery, and intracellular Ca^2+^

**Figure 2.**
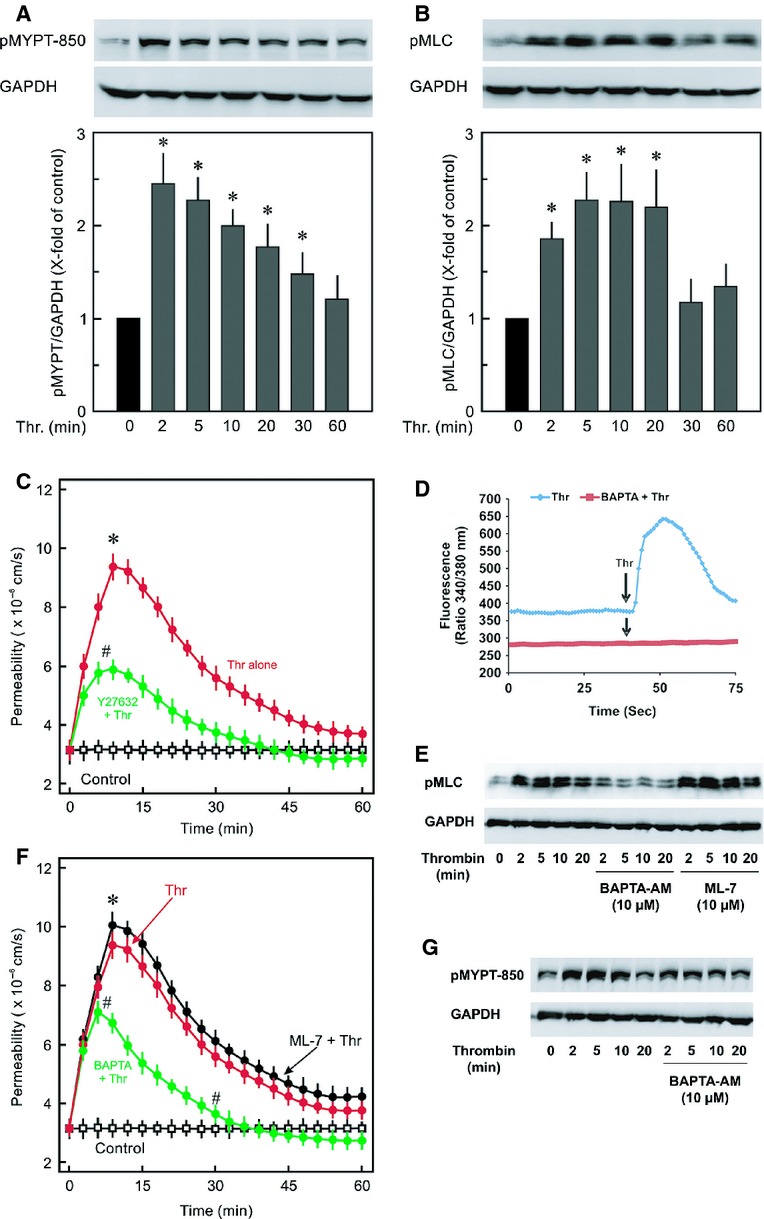
Dynamics of RhoA activity, endothelial contractile machinery, and cytosolic Ca^2+^. EC monolayers were exposed to thrombin (Thr; 0.3 IU/mL) for different time points or vehicle (0 min; control) as indicated and samples were collected in Laemmli buffer and subjected to Western blot analysis for (A) MYPT1 phosphorylation using a phosphospecific antibody to MYPT1 (Thr850) and (B) MLC phosphorylation using a phosphospecific antibody to MLC (S18/T19). GAPDH was used as loading control. Mean ± SEM of three experiments of independent cell preparations; *P < 0.05 versus C (0 min). (C) EC monolayers were exposed to thrombin (Thr; 0.3 IU/mL) in the absence or presence of Rock inhibitor Y27632 (10 *μ*mol/L) or vehicle (control) as indicated and albumin flux (permeability) was measured. Mean ± SEM of three experiments of independent cell preparations; *P <**0.05 versus control, ^#^*P *<**0.05 versus Thr alone. (D) Cytosolic Ca^2+^‐levels (Fura‐2 ratio) was measured as detailed in methods. The graph shows collective mean data of at least 100 cells from one measurement. Representative graph of three experiments from independent cell preparation. (E) MLC phosphorylation. EC monolayers were exposed to thrombin (Thr; 0.3 IU/mL) for different time points or vehicle (0 min; control) in the absence or presence of BAPTA‐AM or ML‐7 as indicated. Representative blots from three experiments with independent cell preparation. (F) EC monolayers were exposed to thrombin (Thr; 0.3 IU/mL) in the absence or presence of ML‐7 (10 *μ*mol/L) or BAPTA‐AM (10 *μ*mol/L) or vehicle (control) as indicated and albumin flux (permeability) was measured. Mean ± SEM of three experiments of independent cell preparations; *P <**0.05 versus control, ^#^*P *<**0.05 versus Thr alone. (G) MYPT1 phosphorylation. EC monolayers were exposed to thrombin (Thr; 0.3 IU/mL) for different time points or vehicle (0 min; control) in the absence or presence of BAPTA‐AM as indicated. Representative blots from three experiments with independent cell preparation.

The actin cytoskeleton is regulated by the members of the Rho family of GTPases, therefore, the changes in activities of RhoA, Rac1, and Cdc42 were analysed. In the first step activation of RhoA/Rock signalling was analysed by measuring the phosphorylation state of MYPT1 at T850 which is directly phosphorylated by Rock and endothelial contractile activation was determined by the phosphorylation state of myosin light chains (MLC). Thrombin caused a 2.5‐fold increase in MYPT1 phosphorylation within 2 min which remained highly phosphorylated for 10 min and declined towards basline after 30 min (Fig. 2A). A similar pattern is observed with phosphorylation of MLC (Fig. 2B). Inhibition of Rock with a pharmacological inhibitor (Y27632; 10 *μ*mol/L) abrogated thrombin‐induced MLC phosphorylation (Aslam et al. 2010) and attenuated thrombin‐induced hyperpermeability (Fig. 2C). The time required for 50% recovery of permeability from the maximum increase was slightly less in the presence of Y27632 (~16.5 min vs. ~15 min, for thrombin alone and thrombin in the presence of Y27632, respectively). Thrombin also induces the release of Ca^2+^ from intracellular stores which can be involved in the activation of endothelial contractile machinery. To understand the mechanism of this contractile activation kinetics of thrombin‐induced Ca^2+^ release were analysed (Fig. 2D) and compared with MLC phosphorylation. The increase in cytoplasmic Ca^2+^ levels is very rapid lasting for 30–60 sec and precedes MLC phosphorylation. Increased cytosolic levels of Ca^2+^ can activate MLCK therefore in the next step role of Ca^2+^ and MLCK in thrombin‐induced contractile activation, hyperpermeability, and EC barrier restoration was analysed. The role of Ca^2+^ was analysed by using a cell permeable Ca^2+^‐chelator (BAPTA‐AM) and MLCK by a specific pharmacological inhibitor (ML‐7). BAPTA‐AM (10 *μ*mol/L) treated cells depicted relatively low basal Fura‐2 signal and thrombin‐induced increase in Fura‐2 signal was completely abrogated (Fig. 2D). Likewise, as shown in Fig. 2E, BAPTA‐AM completely blocked thrombin‐induced increase in MLC phosphorylation, suggesting that thrombin‐induced endothelial contractile activation is Ca^2+^‐dependent. Interestingly, pharmacological inhibition of MLCK could neither abrogate thrombin‐induced MLC phosphorylation nor hyperpermeability (Fig. 2E and F). In contrast, BAPTA‐AM attenuated thrombin‐induced hyperpermeability to similar levels as by Rock inhibitor (Fig. 2F). The time required for 50% recovery of permeability from the maximum increase was significantly less in the presence of BAPTA‐AM (~16.5 min vs. ~10.5 min, for thrombin alone and thrombin in the presence of BAPTA‐AM, respectively). Likewise, BAPTA‐AM also attenuated thrombin‐induced MYPT1 phosphorylation suggesting that thrombin‐induced Rock activation is at least in part mediated via Ca^2+^.

**Figure 3.**
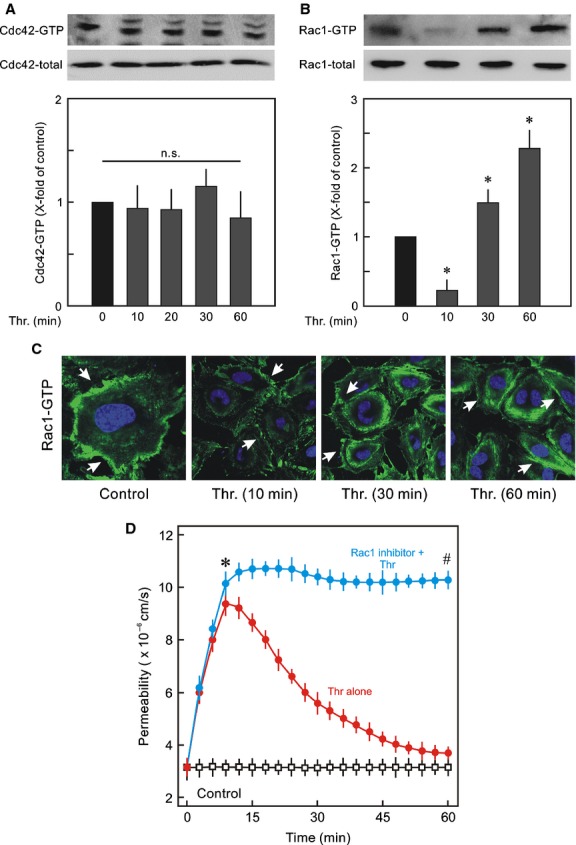
Dynamics of activities of cdc42 and Rac1. (A) Cdc42 activity; Representative Western blots of Rac1‐GTP and Rac1 total. EC monolayers were treated with thrombin (Thr; 0.3 IU/mL) for indicated time points or vehicle (0 min; control) and active Cdc42 was detected by pulldown assay. The active Cdc42 is given as *x*‐fold of control (0 min) taken as 1. Mean ± SEM of three experiments of independent cell preparations; n.s: not significantly different from control. (B) Rac1 activity; Representative Western blots of Rac1‐GTP and Rac1 total. The active Rac1 was detected by pulldown assay. The active Rac1 is given as *x*‐fold of control (0 min) taken as 1. Mean ± SEM of three experiments of independent cell preparations; *P < 0.05. (C) Localisation of active Rac1‐GTP in EC after thrombin treatment. EC monolayers were exposed to thrombin (Thr; 0.3 IU/mL) for different time points (min) as indicated or vehicle (control), PFA fixed, and immunostained for Rac1‐GTP using anti active Rac1 antibody. Representative images of three experiments of independent cell preparation. (D) EC monolayers were exposed to thrombin (Thr; 0.3 IU/mL) in the absence or presence of Rac1 inhibitor NSC23766 (50 *μ*mol/L) or vehicle (control) as indicated and albumin flux (permeability) was measured. Mean ± SEM of three experiments of independent cell preparations; *P <**0.05 versus control, ^#^*P *<**0.05 versus Thr alone.

In the next step activities of other members of Rho family were analysed. Thrombin had no significant effect on cdc42 activation (Fig. 3A), however, it had a biphasic effect on Rac1 activity (Fig. 2B). Thrombin caused a strong inhibition of Rac1 activity in early 10 min and a strong activation of Rac1 occurred after 30–60 min. Similar pattern was seen when active Rac1 was immunostained using specific antibody against active Rac1 (Rac1‐GTP) (Fig. 3C). Rac1‐GTP is localised at cell periphery and ruffles, and exposure of EC to thrombin caused disappearance of active Rac1‐GTP from cell periphery which reappeared after 30 min showing the similar dynamics as observed by pulldown assay (Fig. 3B). Inhibition of Rac1 activation using a pharmacological inhibitor (NSC23766; 50 *μ*mol/L) abrogated barrier recovery (Fig. 3D).

### Effect of actin depolymerisation on VE‐cadherin and RhoA and Rac1 activities

**Figure 4.**
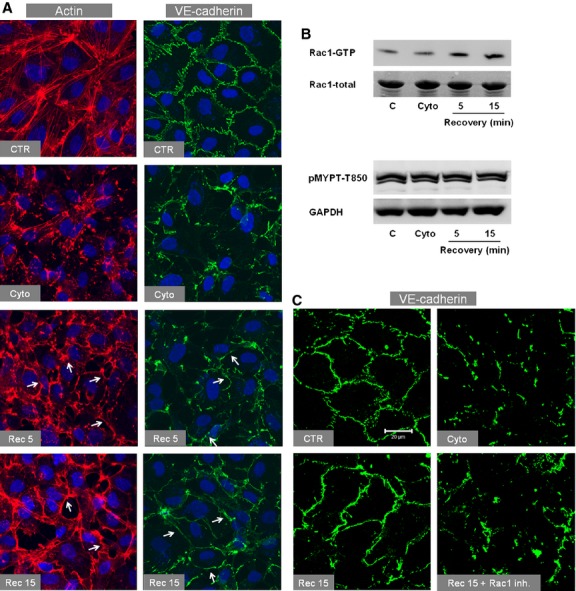
Effect of actin depolymerisation on VE‐cadherin and RhoA and Rac1 activities. (A) Effect of cytochalasin D (cyto) on actin (*left*) and VE‐cadherin (*right*). EC monolayers were treated with cyto (1 *μ*mol/L) or vehicle (CTR; control) for 5 min. After that cyto was washed off and cells were incubated with fresh medium for indicated time points, fixed with paraformaldehyde (actin) or methanol (VE‐cadherin), and immunostained for VE‐cadherin or F‐actin (TRITC‐labelled phalloidin). Arrows denote actin or VE‐cadherin localised at cell borders (representative images of three experiments of independent cell preparations). (B) Effect of cytochalasin D (cyto) on Rac1 (*upper*) and RhoA/Rock signalling (*lower*). Representative Western blots of three experiments of independent cell preparations. EC monolayers were treated with cyto (1 *μ*mol/L) or vehicle (CTR; control) for 5 min. After that cyto was washed off and cells were incubated with fresh medium for indicated time points, cells collected in lysis buffer. Rac1 activation was analysed by pulldown assay while activation of RhoA/Rock signalling was analysed measuring the phosphorylation of MYPT1 at T850. (C) Effect of Rac1 inhibition on reappearance of VE‐cadherin at cell‐cell junctions during the recovery. EC monolayers were treated with cyto (1 *μ*mol/L) or vehicle (CTR; control) for 5 min. After cyto was washed off cells were incubated with fresh medium for 15 min in the absence or presence of Rac1 inhibitor (NSC23766; 50 *μ*mol/L) and cells were immunostained for VE‐cadherin. (Scale bar 20 *μ*m; representative images of three experiments of independent cell preparations). [Rec 5: recovery after 5 min, Rec 15: recovery after 15 min.]

In order to understand the role of RhoA and Rac1 during the re‐establishment of junctions, actin depolymerisation was induced by cytochalasin D (cyto). Cytochalasin D induced complete actin depolymerisation within 5 min (Fig. 4A) accompanied by loss of VE‐cadherin from cell‐cell junctions which was reversible. Immediately after washing off cytochalasin, ECs started re‐establishing the actin polymerisation. Interestingly, the re‐polymerisation of the actin cytoskeleton started at the cell periphery and was accompanied by re‐establishment of cell‐cell junctions which was almost complete within 15 min. In accordance to the pattern of actin re‐polymerisation, a prompt activation of Rac1 but not RhoA/Rock signalling (Fig. 4B) was observed suggesting that in EC Rac1 plays a dominant role in re‐establishing AJs during the recovery process. Inhibition of Rac1 completely abrogated the reappearance of VE‐cadherin at cell‐cell junctions (Fig. 4C).

### Role of cAMP signalling in Rac1 activation and EC barrier restoration

**Figure 5.**
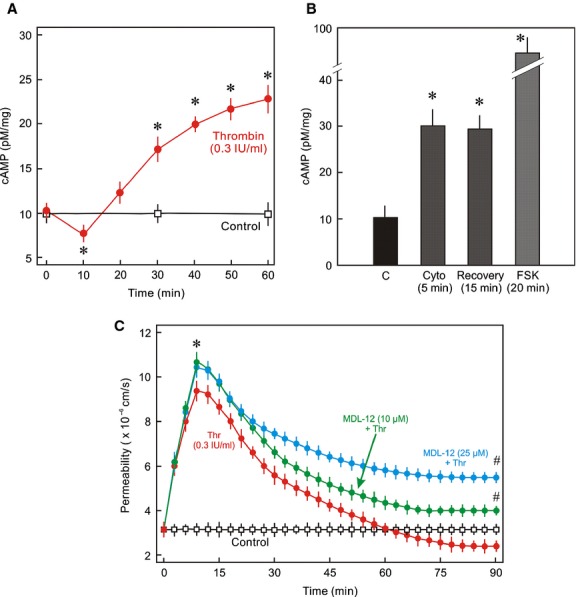
Dynamics of intracellular cAMP levels during EC barrier restoration. (A) Intracellular cAMP levels during the recovery. EC monolayers were treated with thrombin (Thr; 0.3 IU/mL) or vehicle (C; control) for indicated time periods and cAMP levels were measured by ELISA (Mean ± SEM of three experiments of independent cell preparations, *P < 0.05). (B) EC monolayers were treated with cytochalasin D (cyto; 1 *μ*mol/L), forskolin (FSK; 10 *μ*mol/L) or vehicle (C; control) for indicated time points and cAMP levels were measured by ELISA. (Mean ± SEM of three experiments of independent cell preparations, *P < 0.05). (C) Effect of AC inhibition on EC barrier recovery. EC monolayers were exposed to thrombin (Thr; 0.3 IU/mL) in the absence or presence of AC inhibitor MDL12330 (10 and 25 *μ*mol/L) or vehicle (control) as indicated and albumin flux (permeability) was measured. Mean ± SEM of three experiments of independent cell preparations; *P <**0.05 versus control, ^#^*P *<**0.05 versus Thr alone.

**Figure 6.**
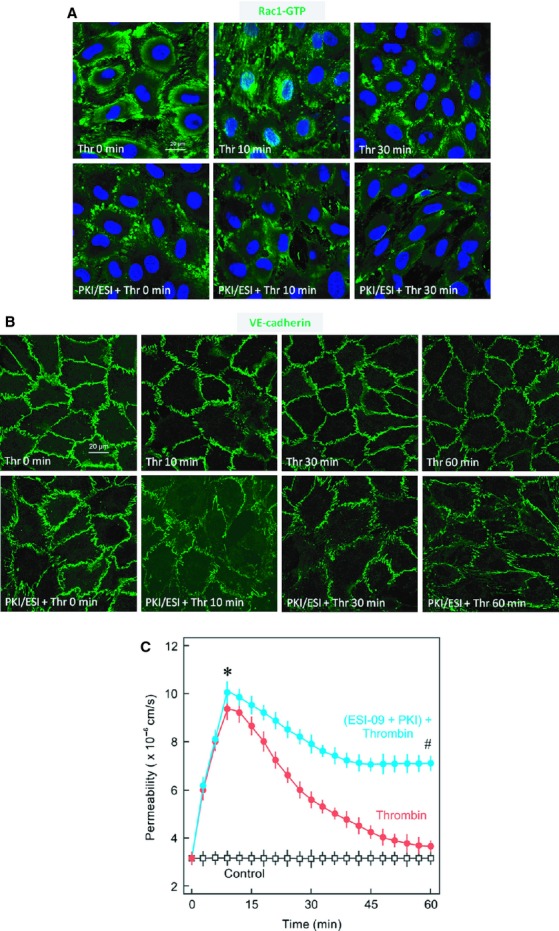
Role of cAMP signalling in Rac1 activation and barrier restoration. (A) Effect of inhibition of cAMP signalling on Rac1 activation. EC monolayers were treated with thrombin (Thr; 0.3 IU/mL) or vehicle (Thr 0 min) for indicated time periods in the absence or presence of PKA and Epac inhibitors (PKI; 10 *μ*mol/L and ESI‐09; 3 *μ*mol/L, respectively) and immunostained for active Rac1 (Rac1‐GTP) using a specific antibody directed against Rac1‐GTP. Nuclei were stained with DAPI. Representative images of Rac1‐GTP from three experiments of independent cell preparation. (B) Effect of inhibition of cAMP signalling on VE‐cadherin localisation. EC monolayers were treated with thrombin (Thr; 0.3 IU/mL) or vehicle (Thr 0 min) for indicated time periods in the absence or presence of PKA and Epac inhibitors (PKI; 10 *μ*mol/L and ESI‐09; 3 *μ*mol/L, respectively) and immunostained for VE‐cadherin using specific antibody directed against VE‐cadherin. Representative images of Rac1‐GTP from three experiments of independent cell preparation. (C) EC monolayers were exposed to thrombin (Thr; 0.3 IU/mL) in the absence or presence of PKA and Epac inhibitors (PKI; 10 *μ*mol/L and ESI‐09; 3 *μ*mol/L, respectively) or vehicle (control) as indicated and albumin flux (permeability) was measured. Mean ± SEM of three experiments of independent cell preparations; *P <**0.05 versus control, ^#^*P *<**0.05 versus Thr alone.

Since the intracellular cAMP is an important regulator of basal activity of Rac1 in ECs, cAMP levels were measured during the EC barrier recovery. Interestingly, thrombin caused a marked reduction in the intracellular cAMP levels which slowly increased during the EC barrier recovery reaching maximum levels after 60 min (Fig. 5A). Likewise, depolymerisation of the actin cytoskeleton by cytochalasin caused an immediate increase in cAMP levels which remained high even after washing off cytochalasin D (Fig. 5B). Forskolin (FSK), a direct activator of the adenylyl cyclase (AC), was used as positive control. Inhibition of AC using a pan inhibitor of AC slowed down the EC barrier recovery (Fig. 5C). cAMP, downstream activates two main effectors, PKA and Epac, which mediate its effect on EC barrier function. Combined pharmacological inhibition of both PKA and Epac by PKI (10 *μ*mol/L) and ESI‐09 (3 *μ*mol/L), respectively, abrogated activation of Rac1 (Fig. 6A), re‐established AJs (Fig. 6B), and EC barrier recovery (Fig. 6C).

## Discussion

The precise regulation of the semi‐selective barrier function of VE is very important for the exchange of nutrients and metabolic products between the blood and the interstitium. Inflammatory mediators such as thrombin induce a transient reversible disruption of EC barrier function which is reversible. However, the molecular mechanisms regulating EC barrier recovery/restoration are incompletely understood. The present study focused on the dynamics of contractile activation and AJs assembly and disassembly during the process of thrombin‐induced EC barrier disruption and restoration.

Thrombin causes a transient increase in EC permeability showing maximum effect after 10 min which thereafter declines towards basal level and is completely restored after 60–90 min. The barrier disrupting signals such as increased intracellular Ca^2+^ levels and EC contractile machinery and RhoA/Rock signalling are activated during this early period (10 min) of hyperpermeability. However, during the barrier recovery phase these signals are inactivated. In order to understand whether these signalling pathways themselves play a role in the EC barrier restoration process, these pathways were pharmacologically intervened. Inhibition of RhoA/Rock signalling with Y27632 and chelating intracellular Ca^2+^ attenuated thrombin‐induced increase in EC permeability to a similar extent but no gross changes in the rate of EC barrier recovery were observed with Y27632 though Ca^2+^ chelation significantly enhanced the rate of EC barrier recovery. Interestingly, thrombin‐induced MLC phosphorylation was completely blocked by Ca^2+^‐chelation but not by a MLCK inhibitor (ML‐7), suggesting that MLCK does not play a major role in thrombin‐induced contractile activation in HUVEC although it antagonised hypoxia/reoxygenation‐induced EC hyperpermeability (Aslam et al. 2013). In line to these results, ML‐7 could neither attenuate the thrombin‐induced increase in EC permeability nor enhance the barrier recovery. These data demonstrate that MLCK does not play any role in thrombin‐induced increase in EC permeability. A similar report shows that MLCK‐dependent contraction is not involved in platelet aggregation factor (PAF)‐induced venular hyperpermeability (Adamson et al. 2003). However, in contrast to the present report, Moy et al. demonstrated that thrombin‐induced hyperpermeability in HUVEC was blocked by ML‐7 (Moy et al. 2002). The discrepancy between ours and this report is possibly due to high concentrations of drugs used by Moy group. They used thrombin at a very high concentration (7 U/mL) which is more than 20‐fold used in the present study and ML‐7 was used at concentration of 100 *μ*mol/L (10‐fold higher compared to the present study). ML‐7 at high concentration may also inhibit PKC (Odani et al. 2003) and these inhibitory effects reported by Moy group are possibly due to inhibition of PKC and not MLCK.

The thrombin‐induced MYPT1 phosphorylation was attenuated by Ca^2+^‐chelator suggesting that Ca^2+^ plays an important role in thrombin‐induced activation of RhoA/Rock signalling and contractile machinery. How precisely Ca^2+^ activates RhoA/Rock signalling is not well understood. However, it has been reported that thrombin activates p115RhoGEF in a PKC*α*‐dependent manner (Kozasa et al. 1998; Holinstat et al. 2003), suggesting that Ca^2+^‐mediated activation of RhoA/Rock may be via PKC*α*‐induced activation of p115RhoGEF.

Next the dynamics of changes in the activities of cdc42 and Rac1 were analysed. Although, a delayed activation of cdc42 in human dermal microvascular EC line (HMEC‐1) 60 min after the thrombin challenge has been reported (Kouklis et al. 2004), no change in cdc42 activity within the first 60 min after thrombin was observed in primary HUVEC monolayers in the present study. Moreover, the barrier recovery process after thrombin challenge starts much earlier than 60 min when the resealing is already almost complete. In the present study, a strong inhibition of Rac1 activity was observed after 10 min which is hyper‐activated after 30–60 min. Rac1 is critical for the maintenance of basal endothelial permeability (Waschke et al. 2004a) and promotes AJs stability via actin cytoskeleton remodelling at the cell periphery and providing a support to AJ complexes (Schnittler et al. 2014). Indeed, the cytochalasin experiments demonstrate clearly that it is Rac1 but not RhoA which regulates the reorganisation of actin at cell periphery and re‐establishment of AJs after injury.

The activity of Rac1 in ECs is modified by intracellular cAMP levels (Waschke et al. 2004a,b) and certain basal cAMP levels are required for the maintenance of endothelial barrier function (Spindler and Waschke 2011). We demonstrate that indeed thrombin causes a reduction in cAMP levels during the early time period, which are raised gradually higher than the basal levels during the recovery phase. Likewise, breakdown of actin cytoskeleton with cytochalasin also resulted in increased levels of intracellular cAMP suggesting that it is a universal protective mechanism of ECs for actin and AJs remodelling. Inhibition of AC using a pan AC inhibitor slowed down the barrier recovery after thrombin challenge confirming the role of cAMP in EC barrier recovery.

In the present study the molecular mechanism of cAMP production was not investigated. Thrombin induces the release of ATP from HUVEC in Ca^2+^‐dependent manner (Gödecke et al. 2012) which can be hydrolysed to adenosine and thus may induce cAMP production via adenosine A2 receptors. However, in the present study, we were unable to observe any significant effect of adenosine A2 receptor antagonists (DMPX and ZM‐241,385) on the kinetics of EC barrier recovery (data not shown) excluding the involvement of this pathway. Thrombin may also cause activation of AC6 in ECs via Ca^2+^‐ and PLA‐dependent production of prostacycline in a paracrine manner (Werthmann et al. 2011), and an inhibition of cytosolic Ca^2+^ rise with BAPTA and cyclooxygenase with indomethacin attenuates cAMP production by thrombin (Werthmann et al. 2011) and abrogated AC6 overexpression‐mediated EC barrier protection (Bundey and Insel 2006). This suggests a dual role of Ca^2+^ in thrombin‐induced EC barrier disruption. Firstly, it induces the activation of RhoA/Rock and PKC signalling leading to contractile activation and barrier failure; secondly, it induces cAMP production which starts the recovery of EC barrier function. If this is the case, inhibition of cytosolic Ca^2+^‐rise should abrogate the EC barrier recovery, but the present study demonstrates that inhibition of Ca^2+^ rise actually protects EC barrier function and fastens the recovery suggesting the mechanism of cAMP production and involvement of Ca^2+^ is complex and needs further investigation.

The cAMP downstream activates two effector proteins, the PKA and Epac. (Walsh et al. 1968; de Rooij et al. 1998; Aslam et al. 2010) Both of these effectors downstream activate Rac1 (Birukova et al. 2008, 2010; Aslam et al. 2012, 2013). Combined inhibition of both PKA (with a cell permeable peptide inhibitor PKI) (Lum et al. 1999) and Epac (using a pharmacological inhibitor ESI‐09) (Almahariq et al. 2013; Gong et al. 2013) abrogated Rac1 activation during the recovery phase and re‐establishment of AJs and EC barrier recovery. In line to these findings, recently Rap1 has been shown to be involved in the EC barrier recovery (Birukova et al. 2013). Rap1 is a direct target of Epac and downstream activates Rac1 (Birukova et al. 2010).

In conclusion we demonstrate that cAMP is an important factor controlling the re‐establishment of endothelial AJs and recovery of EC barrier function after thrombin challenge. cAMP signalling activates Rac1 which regulates the remodelling of actin cytoskeleton at cell periphery and stabilises AJs. Cytosolic Ca^2+^ concentrations are important triggers in inducing EC barrier disruption but not recovery and inhibition of Ca^2+^ rise enhances the rate of EC barrier recovery. Importantly, MLCK neither play significant role in thrombin‐induced barrier failure nor barrier recovery.

## Acknowledgments

The technical support by S. Schäffer, D. Reitz, H. Thomas and A. Reis is gratefully acknowledged. All experiments were performed at the Department of Cardiology and Angiology, University Hospital, Giessen, and the Institute of Physiology Justus Liebig University, Giessen, Germany.

## Conflict of Interest

None declared.
